# Estrogenic Activity of 4-Hydroxy-Benzoic Acid from *Acer tegmentosum* via Estrogen Receptor α-Dependent Signaling Pathways

**DOI:** 10.3390/plants11233387

**Published:** 2022-12-05

**Authors:** Quynh Nhu Nguyen, Seoung Rak Lee, Baolo Kim, Joo-Hyun Hong, Yoon Seo Jang, Da Eun Lee, Changhyun Pang, Ki Sung Kang, Ki Hyun Kim

**Affiliations:** 1 College of Korean Medicine, Gachon University, Seongnam 13120, Republic of Korea; 2 School of Pharmacy, Sungkyunkwan University, Suwon 16419, Republic of Korea; 3 School of Chemical Engineering, Sungkyunkwan University, Suwon 16419, Republic of Korea

**Keywords:** *Acer tegmentosum*, 4-hydroxy-benzoic acid, phytoestrogens, estrogen receptor

## Abstract

*Acer tegmentosum*, a deciduous tree belonging to Aceraceae, has been used in traditional oriental medicine for treating hepatic disorders, such as hepatitis, cirrhosis, and liver cancer. We evaluated the estrogen-like effects of *A. tegmentosum* using an estrogen receptor (ER)-positive breast cancer cell line, namely MCF-7, to identify potential phytoestrogens and found that an aqueous extract of *A. tegmentosum* promoted cell proliferation in MCF-7 cells. Five phenolic compounds (**1**–**5**) were separated and identified from the active fraction using bioassay-guided fractionation of crude *A. tegmentosum* extract and phytochemical analysis. The chemical structures of the compounds were characterized as vanillic acid (**1**), 4-hydroxy-benzoic acid (**2**), syringic acid (**3**), isoscopoletin (**4**), and (*E*)-ferulic acid (**5**) based on the analysis of their nuclear magnetic resonance spectra and liquid chromatography-mass spectrometry data. All five compounds were evaluated using an E-screen assay for their estrogen-like effects on MCF-7 cells. Among the tested compounds, only 4-hydroxy-benzoic acid (**2**) promoted the proliferation of MCF-7 cells, which was mitigated by the ER antagonist, ICI 182,780. The mechanism underlying the estrogen-like effect of 4-hydroxy-benzoic acid (**2**) was evaluated via western blotting analysis to determine the expression levels of extracellular signal-regulated kinase (ERK), phosphoinositide 3-kinase (PI3K), serine/threonine kinase (AKT), and ERα. Our results demonstrated that 4-hydroxy-benzoic acid (**2**) induced the increase in the protein expression levels of p-ERK, p-AKT, p-PI3K, and p-Erα, concentration dependently. Collectively, these experimental results suggest that 4-hydroxy-benzoic acid (**2**) is responsible for the estrogen-like effects of *A. tegmentosum* and may potentially aid in the control of estrogenic effects during menopause.

## 1. Introduction

Menopause, the permanent ending of the menstrual cycle, typically occurs in women aged 45–55 years. With increasing life expectancy, menopausal women aged ≥50 years worldwide are estimated to reach 1.2 billion by 2030, along with 47 million new entrants each year [1,2]. The Korean postmenopausal female population aged >50 years has increased since 2000, and over half of the female population is estimated to be postmenopausal by 2030, based on the Korean Statistical Information Service database [3]. The menopause is known to be caused by the decline of ovarian function, where the production of estrogen is drastically decreased. The menopause causes vasomotor symptoms, such as night sweats, sleep disturbance, hot flushes, vaginal dryness, and osteoporosis [4]. Women experiencing menopausal symptoms have complained about significantly low health-related quality of life, and exogenous estrogens are prescribed to women in severe cases [5].

Estrogen replacement therapy (ERT) is a beneficial option for the relief and prevention of menopausal symptoms and related diseases [6,7]. However, patients taking long-term ERT are often reluctant to continue owing to severe side effects, such as heart disease, breast cancer, and stroke [8,9]. Accordingly, phytoestrogens with estrogen-like activity have been attractive. Phytoestrogens are compounds naturally found in plants and plant-derived foods, and they possess similar functionality to mammalian estrogens or their active metabolites due to their similar structure [10]. For examples, phytoestrogens, including isoflavonoids, flavonoids, stilbenes, and lignans, are known to be abundant in red clover and soy plants, and flax seeds [11]. The active site for phytoestrogens has been reported to be the estrogen receptor (ER), and phytoestrogens act as agonists or antagonists by binding to ER [12]. As they have similar structures to estrogen, they are sometimes reported to increase the risk of breast cancer [7]. However, many studies on breast cancer relating to phytoestrogens have reported that they do not exhibit any effects on cancers [13].

*Acer tegmentosum* (*Aceraceae*) is a deciduous tree widely found in East Asia, including Korea and East China, and is widely used in folk medicine as a remedy for hepatic disorders, such as cirrhosis, hepatitis, and liver cancer [14]. With growing experimental evidence of its pharmacological effects, including antioxidant, neuro- and hepato-protective, and anti-inflammatory effects, *A. tegmentosum* has been gradually incorporated as a health food and was officially accepted as a functional food in 2014 by the Korean Food and Drug Administration (KFDA) [15]. Hepatoprotective effects of *A. tegmentosum* can be used to mitigate pathological symptoms, such as liver fibrosis, obesity, cancer, depression, and osteoporosis [16,17]. A previous phytochemical study of this plant reported some bioactive small molecules, including lignans, flavonoids, and phenolic compounds [17,18,19].

Our group has been conducting extensive natural product research work to discover bioactive phytochemicals from various natural resources [20,21,22,23,24,25]. In this context, we investigated the potential bioactive phytochemicals from an extract of *A. tegmentosum*. A previous study by our group on *A. tegmentosum* led to the identification of bioactive flavonoids and phenolic compounds that exhibited preventive effects against oxidative stress in dopaminergic neurons and alcohol-induced steatosis in the liver [26,27]. In addition, our group reported the presence of isoamericanoic acid B as a promising phytoestrogen in *A. tegmentosum* [28]. In our ongoing research on *A. tegmentosum,* we found that an aqueous extract of *A. tegmentosum* exhibited estrogen-like effects in an ER-positive MCF-7 breast cancer cell line. These estrogen-like effects of *A. tegmentosum* have not been previously reported outside of our group. Therefore, the current study was conducted to further explore the active extracts of *A. tegmentosum* to identify potential phytoestrogens using bioactivity-guided fractionation. Herein, we performed the isolation and structural characterization of five compounds, evaluated their estrogen-like effects on MCF-7 cells, and characterized their bioactivity as phytoestrogens.

## 2. Results

### 2.1. Bioactivity-Guided Fractionation of the Aqueous Extract of A. tegmentosum

**Figure 1:**
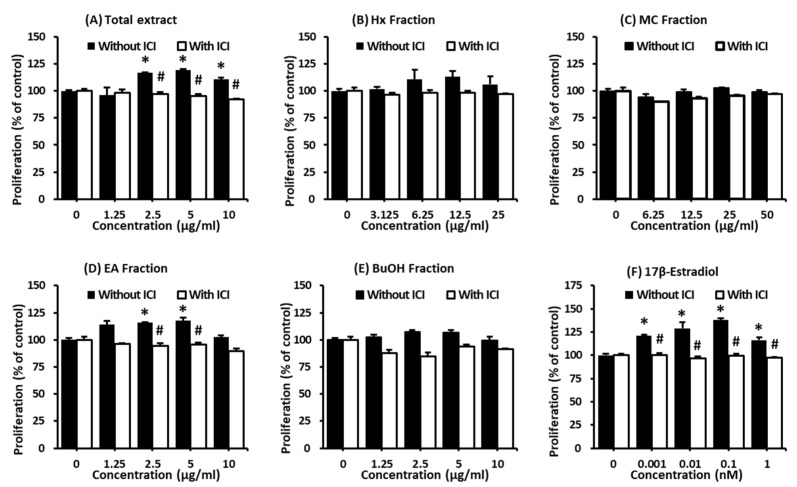
Comparison of the estrogenic activity of the (**A**) crude extract (total); (**B**) hexane (Hx), (**C**) CH_2_Cl_2_ (MC), (**D**) EtOAc (EA), and (**E**) *n*-BuOH (BuOH) fractions; and (**F**) 17β-estradiol in the absence or presence of ICI 182,780 according to the cell proliferation measured by E-screen assay in MCF-7 cells. * Significant difference between the cells treated with the extract or fractions and untreated cells. # Significant difference between the cells treated with ICI and without ICI at the same concentration of sample (*n* = 3 independent experiments, *p* < 0.05, Kruskal–Wallis non-parametric test). Data are represented as the mean ± standard error of the mean (SEM).

**Figure 2:**
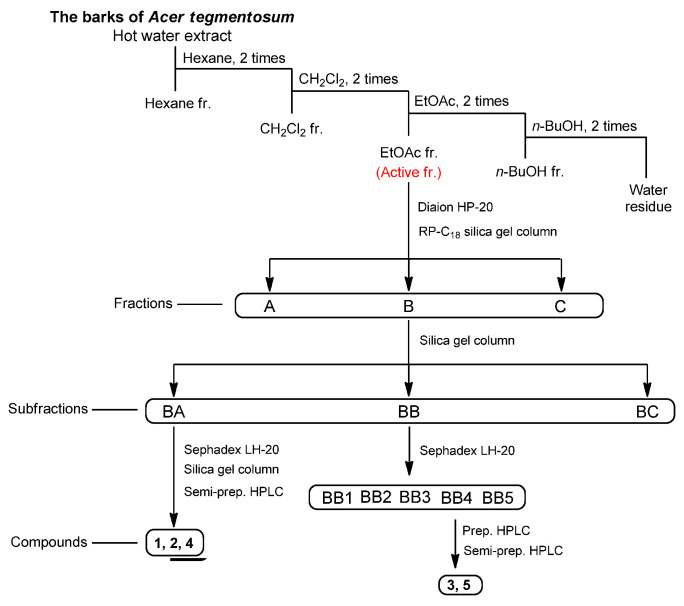
Isolation scheme of compounds **1**–**5** from the extract of *Acer tegmentosum*.

MCF-7 cell proliferation was tested after treatment with the aqueous extract of *A. tegmentosum* using Ez-Cytox reagent. The MCF-7 cell proliferation increased to 119.1 ± 0.2% after treatment with 5 µg/mL of the extract compared to that in untreated cells, while the effect was mitigated by the ER antagonist, ICI 182,780 (Figure 1A). According to the result, the crude extract was successively solvent-partitioned by using hexane, EtOAc, CH_2_Cl_2_, and *n*-BuOH to obtain four main solvent-partitioned fractions (Figure 2). To perform the bioactivity-guided fractionation, the estrogen-like effects of the four solvent-partitioned fractions were evaluated by using a cell proliferation assay to confirm the active fraction. Among the fractions tested, treatment with 5 µg/mL of the EA fraction increased the cell proliferation to 117.6 ± 1.9% compared to that in the untreated cells, and the increased effect was mitigated by ICI 182,780 (Figure 1D), which indicated that the active fraction may be the EA fraction.

### 2.2. Isolation of Compounds from the Active Fraction and Their Identification

**Figure 3:**
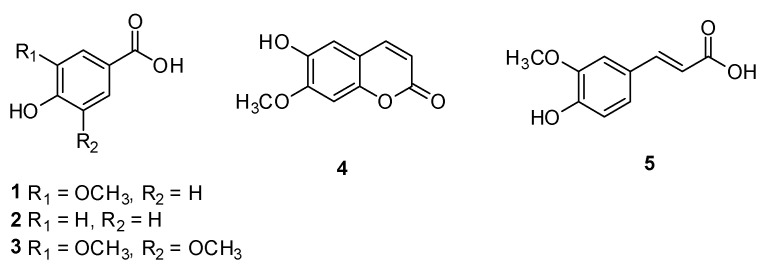
Chemical structures of compounds **1–6**: vanillic acid (**1**), 4-hydroxy-benzoic acid (**2**), syringic acid (**3**), isoscopoletin (**4**), and (*E*)-ferulic acid (**5**).

According to the results of bioactivity-guided fractionation regarding estrogen-like effects, a phytochemical analysis of the active EtOAc fraction was carried out to identify the active compounds to the estrogenic activity of *A. tegmentosum* extract. Chemical investigation of the EtOAc fraction applying open column chromatography and preparative and semi-preparative high-performance liquid chromatography (HPLC) purification as well as thin-layer chromatography (TLC) analysis led to the isolation and identification of five phenolic compounds (**1**–**5**; Figure 2). The isolated compounds were determined to be vanillic acid (**1**) [29], 4-hydroxy-benzoic acid (**2**) [30], syringic acid (**3**) [31], isoscopoletin (**4**) [32], and (*E*)-ferulic acid (**5**) [33] (Figure 3) based on the analysis of their nuclear magnetic resonance (NMR) spectra and comparison with previously reported data in the literature as well as LC/MS data.

### 2.3. Effects of Compounds on the Proliferation of MCF-7 Cells

**Figure 4:**
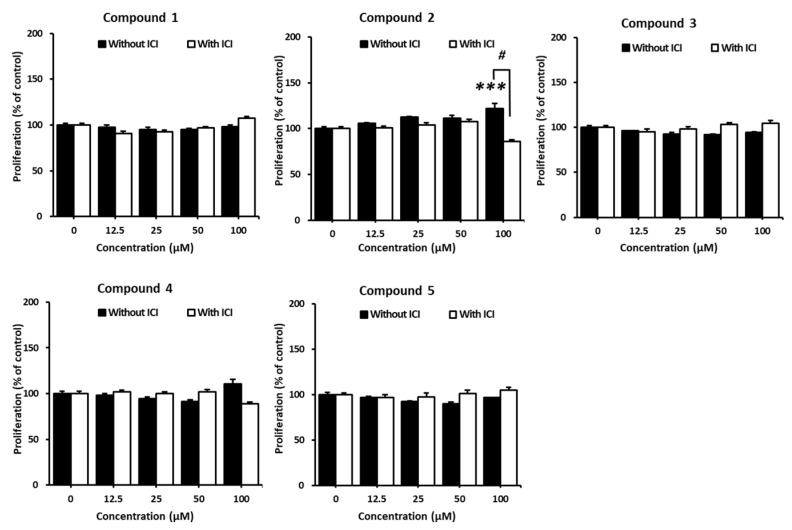
Estrogenic effects of the isolated compounds (**1–5**) in the presence or absence of ICI 182,780 measured by the E-screen assay on MCF-7 cells. Cell viability is expressed as the mean ± SEM. *** *p* < 0.001 compared to the non-treated group; ^#^ *p* < 0.05 compared to the ICI-treated group at the same concentration of samples. Data are represented as the mean ± SEM.

The compounds (**1–5**) isolated in this study were tested for their proliferation effects on MCF-7 cells to investigate their potential estrogenic activity. As a result, only 4-hydroxy-benzoic acid (**2**) showed the increased proliferation of MCF-7 cells. Cell proliferation increased significantly to 121.9 ± 5.63% after treatment with 100 µM of 4-hydroxy-benzoic acid (**2**), and the effects were controlled by ICI 182,780 as an ER antagonist (Figure 4). These results suggest that 4-hydroxy-benzoic acid (**2**) may be a potential phytoestrogen, exhibiting estrogen-like effects on ER-positive breast cancer cells.

### 2.4. Effect of 4-Hydroxy-Benzoic Acid on the Protein Expression Levels of p-Phosphoinositide 3-Kinase (p-PI3K), PI3K, Phospho-Serine/Threonine Kinase (p-AKT), AKT, p-ERα, and ERα

**Figure 5:**
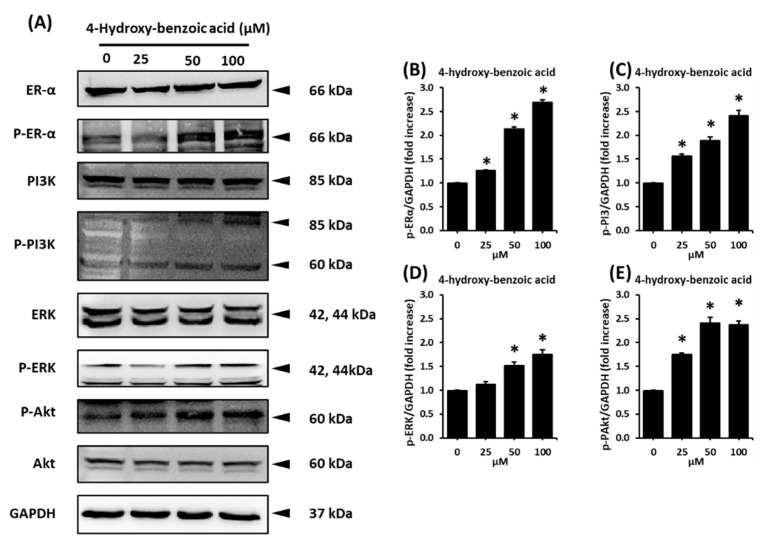
(**A**) Effects of 4-hydroxy-benzoic acid (**2**) on the protein expression levels of p-ERK, ERK, p-PI3K, PI3K, p-AKT, AKT, p-ERα, ERα, and GAPDH in MCF-7 cells treated or untreated with 25, 50, and 100 µM 4-hydroxy-benzoic acid (**2**) for 96 h. (**B**–**E**) The bar graph presents the densitometric quantification of the western blot bands. * Significant difference between the treated cells with 4-hydroxy-benzoic acid (**2**) and untreated cells. Significant difference between the treated cells with ICI and without ICI at the same concentration of the sample (*n* = 3 independent experiments, *p* < 0.05, Kruskal–Wallis non-parametric test) (*n* = 3 independent experiments, *p* < 0.05, Kruskal–Wallis non-parametric test). Data are represented as the mean ± SEM.

To confirm the mechanism of action of 4-hydroxy-benzoic acid (**2**) on the proliferation of MCF-7 cells, the expression of ERα and related pathways was investigated using western blotting analysis. Compared to the untreated cells, 25, 50, and 100 µM of 4-hydroxy-benzoic acid (**2**) induced the increase concentration dependently in the protein expression levels of phospho-extracellular signal-regulated kinase (p-ERK), p-PI3K, p-AKT, and p-ERα, whereas the levels of ERK, PI3K, ER-α, as well as glyceraldehyde 3-phosphate dehydrogenase (GAPDH) as the housekeeping gene, remained unchanged (Figure 5A). Protein expression ratio between p-ERK, p-AKT, p-PI3K, and p-ERα increased significantly in a dose-dependent manner after treatment with 4-hydroxy-benzoic acid (Figure 5B–E). The protein expression of p-ERα had the highest ratio at 2.1 ± 0.04 and 2.7 ± 0.05-folds of GAPDH at concentrations of 50 and 100 µM, respectively.

## 3. Discussion

**Figure 6:**
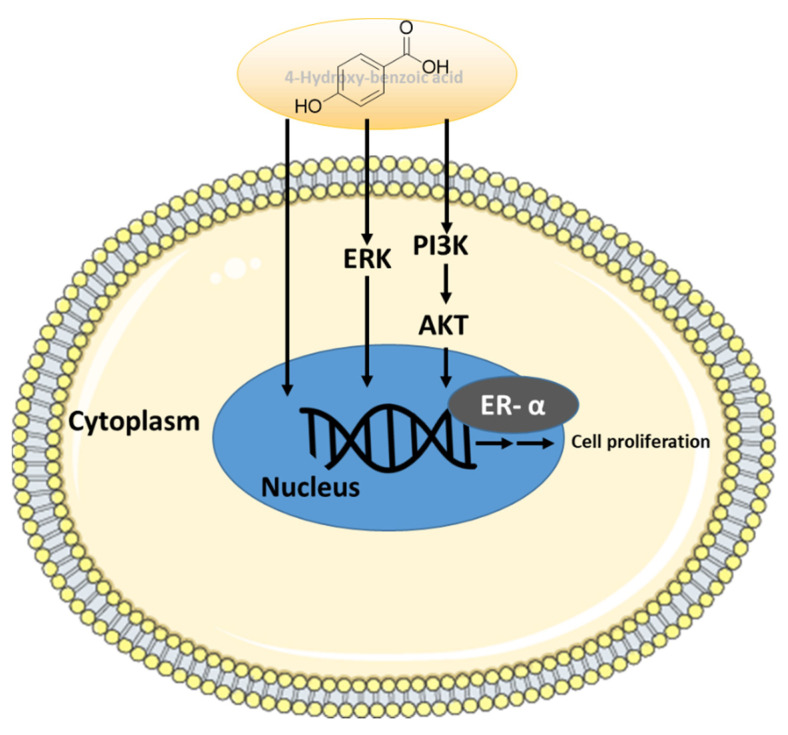
Schematic illustration of the mechanism underlying the estrogenic activity regarding to 4-hydroxy-benzoic acid via ERα-dependent signaling pathways in the ER-positive breast cancer cells (MCF-7).

*Acer* species have been studied for their anti-angiogenic, anti-atopic, and antioxidant effects [34]. *A. tegmentosum* has shown ethnopharmacological capacity against hepatic inflammation in vitro and in vivo [14]. The hepatoprotective effects from *A. tegmentosum* extract have been reported to be associated with its antioxidant activity, together with the regulation of autophagy [15]. The MeOH extract from the *A. tegmentosum* twigs contains 10 phenolic compounds, which was determined to exhibit cytotoxic activity against several cancer cell lines [35]. Our previous study of *A. tegmentosum* reported a potential phytoestrogen, isoamericanoic acid B, using docking simulation, as it showed good estrogenic activity with ERα and ERβ [28]. Extending our previous study, the current work found that the EtOAc fraction of *A. tegmentosum* extract displayed estrogen-like effects on MCF-7 cells. Five compounds were identified from the active EtOAc fraction, and only 4-hydroxy-benzoic acid (compound **2**) promoted the cell proliferation of MCF-7 cells. Its co-treatment with ICI 182,780, which is an ER antagonist, inhibited this proliferation-stimulating effect. These results indicate that compound **2** exhibits a proliferation-stimulating effect through ER in MCF-7 cells, which was also confirmed by the protein expression involved in the ER-signaling pathway. Binding of estrogen to the G-protein-coupled ER was reported to activate the ERK and PI3K/AKT pathways [36,37]. ERK is known as one of the family of mitogen-activated protein kinases, which can be stimulated by peptide hormones and cytokines, sometimes cellular stress. It has been known to regulate the proliferation of ER-positive breast-cancer cells [38]. In addition, in normal estrogen-responsive tissues, the activated PI3K/AKT pathway is reported to regulate the cell growth, survival, as well as proliferation [39,40]. Various previous studies have revealed the estrogen-like effect of acacetin, which is one of the flavonoids, and its possible mechanism was determined to be the ERK and PI3K/AKT pathways [41,42,43]. In our study, 4-hydroxy-benzoic acid was found to mediate the dose-dependent increase in the protein expression levels of p-ERK, p-AKT, p-Erα, and p-PI3K in MCF-7 cells (Figure 6), similar to the other reported phytoestrogens. Therefore, the estrogen-like effect of 4-hydroxy-benzoic acid is mainly mediated by ERα.

Several previous studies have shown the estrogenic activity of 4-hydroxy-benzoic acid both in vitro with human cancer cell lines [44] and in vivo with a CD1 mouse model [45]. However, these studies did not verify the mechanism of action for its estrogenic activity. The mechanistic studies of this study confirmed that 4-hydroxy-benzoic acid exhibits potent estrogen-like effects as mentioned above. In addition, in our previous study, we found that isoamericanoic acid B could be a potential phytoestrogen [28]. Isoamericanoic acid B was isolated from subfraction BC, which is one of the subfractions of the active EtOAc fraction, whereas our current compounds were extracted from subfractions BA and BB. Therefore, our compounds together with isoamericanoic acid B might also contribute to the estrogenic activity of the EtOAc fraction of *A. tegmentosum*. Our data suggest that *A. tegmentosum* should be studied further in animals to find other phytoestrogenic compounds capable of reducing the adverse effects of changes in postmenopausal women.

## 4. Materials and Methods

### 4.1. General Experimental Procedure

Detailed information is included in the Supplementary Materials.

### 4.2. Plant Material

Detailed information is included in the Supplementary Materials.

### 4.3. Extraction and Isolation

*A. tegmentosum* bark was dried and crushed to acquire 2 kg of material. The bark was extracted using distilled water (1 L) at 90 °C for 10 h and filtered with Whatman No 1 filter papers. The filtrate was evaporated *in vacuo* to obtain a brownish extract (232 g), which was dissolved in distilled water (700 mL × 3). The dissolved extract was solvent-partitioned twice using hexane, CH_2_Cl_2_, EtOAc, and *n*-BuOH to yield 62.1, 32.2, 22.4, and 37.3 g, respectively. EtOAc-soluble fraction was subjected to a Diaion HP-20 chromatography with 500 mL of each solvent system of 20, 40, 60, 80, and 100% MeOH in H_2_O. The 80% and 100% MeOH soluble fractions were combined into one fraction (4.2 g) and successively fractionated using RP-C_18_ silica gel (230–400 mesh) column chromatography with a gradient solvent system of MeOH–H_2_O (1:1 to 1:0, *v/v*) to obtain three fractions (A–C). Fraction B (3.3 g) was loaded onto a silica gel (230–400 mesh) column and eluted with a gradient solvent system of EtOAc–MeOH (30:1 to 1:1, *v/v*) to yield three fractions (BA–BC). Fraction BA (511 mg) was separated via Sephadex LH-20 column chromatography using 100% MeOH to obtain three fractions (BA1–3). Three subfractions (BA21–23) were acquired from fraction BA2 (187 mg) using a silica gel (70–230 mesh) chromatography column and eluted with a gradient solvent system of EtOAc–MeOH (50:1 to 5:1, *v/v*). Subfraction BA22 (62 mg) was purified via semi-preparative reverse-phase HPLC with an isocratic solvent system of 28% MeOH (flow rate: 2 mL/min) to yield compounds **1** (7.3 mg, *t*_R_ = 45.0 min), **2** (4.6 mg, *t*_R_ = 55.0 min), and **4** (7.6 mg, *t*_R_ = 58.0 min). Fraction BB (564 mg) was separated via Sephadex LH-20 column chromatography and eluted with 100% MeOH to obtain five fractions (BB1–BB5). Fraction BB4 (136 mg) was separated via preparative reverse-phase HPLC with a gradient solvent system of MeOH–H_2_O (3:7 to 4:6, flow rate: 5 mL/min) to provide four subfractions (BB41–BB44). Compound **3** (8.1 mg, *t*_R_ = 33.0 min) was purified from subfraction BB41 (15 mg) via semi-preparative reverse-phase HPLC with an isocratic solvent system of 30% MeOH (flow rate: 2 mL/min). Subfraction BB44 (12 mg) was purified via semi-preparative reverse-phase HPLC and eluted with an isocratic solvent system of 36% MeOH (flow rate: 2 mL/min) to obtain compound **5** (3.9 mg, *t*_R_ = 50.0 min).

### 4.4. Cell Culture

The detailed information is included in the Supplementary Materials.

### 4.5. E-Screen Assay

MCF-7 cells were inoculated into a 96-well plate at a density of 7.5 × 10^3^ cells in 100 μL per well with 95% relative humidity, 5% CO_2_, and 37 °C. After incubating for 24 h, the seeding medium was removed and replaced by sample treatment in phenol red-free RPMI medium supplemented with 10% charcoal-stripped heat-inactivated human serum (Innovative Research, Novi, MI, USA), 100 U/mL penicillin, and 100 μg/mL streptomycin (Gibco BRL, Carlsbad, MD, USA). The treatment was continued for 96 h, and cell proliferation was measured using a 10% Ez-Cytox cell proliferation assay kit (Daeil Lab Service Co., Seoul, Republic of Korea) in culture medium for 1 h using a microplate reader (PowerWave XS; Bio-Tek Instruments, Winooski, VT, USA) [46].

### 4.6. Western Blotting Analysis

Detailed information is included in the Supplementary Materials.

## 5. Conclusions

In this study, we identified the estrogenic effects of 4-hydroxy-benzoic acid (**2**), isolated from the extract of *A. tegmentosum*, on MCF-7 ER-positive breast cancer cells using bioassay-guided fractionation. Our results demonstrated that 4-hydroxy-benzoic acid (**2**) significantly enhanced the proliferation of MCF-7 cells, which was determined to be associated with the activation of ERK, AKT, PI3K, and ERα. Taken together, our findings suggest that 4-hydroxy-benzoic acid (**2**) can be a potential phytoestrogen for the development of natural estrogen supplements.

## Supplementary Materials

The following supporting information can be downloaded at: https://www.mdpi.com/article/10.3390/plants11233387/s1, Figure S1: ^1^H NMR spectrum of **1** (CD_3_OD, 800 MHz), Figure S2: ^13^C NMR spectrum of **1** (CD_3_OD, 200 MHz), Figure S3: HPLC chromatogram and UV and MS data of LC/MS for compound **1**, Figure S4: ^1^H NMR spectrum of **2** (CD_3_OD, 800 MHz), Figure S5: HPLC chromatogram and UV and MS data of LC/MS for compound **2**, Figure S6: ^1^H NMR spectrum of **3** (CD_3_OD, 800 MHz), Figure S7: ^13^C NMR spectrum of **3** (CD_3_OD, 200 MHz), Figure S8: HPLC chromatogram and UV and MS data of LC/MS for compound **3**, Figure S9: ^1^H NMR spectrum of **4** (CD_3_OD, 800 MHz), Figure S10: HPLC chromatogram and UV and MS data of LC/MS for compound **4**, Figure S11: ^1^H NMR spectrum of **5** (CD_3_OD, 800 MHz), and Figure S12: HPLC chromatogram and UV and MS data of LC/MS for compound **5**.
