# Supplementary material for: Estrogenic Activity of 4-Hydroxy-Benzoic Acid from Acer tegmentosum via Estrogen Receptor α-Dependent Signaling Pathways
Source: Plants (Basel). 2022 Dec 5;11(23):3387. doi: 10.3390/plants11233387 (PMC9740217; doi:10.3390/plants11233387)
Supplement: Supplementary file 1 [file plants-11-03387-s001.zip › plants-2005550-supplementary.pdf]

Supplementary data

Estrogenic Activity of 4-Hydroxy-Benzoic Acid from *Acer tegmentosum* via Estrogen Receptor α-Dependent Signaling Pathways

Quynh Nhu Nguyen [1,†], Seoung Rak Lee [2,†], Baolo Kim [1,†], Joo-Hyun Hong [2], Yoon Seo Jang [2], Da Eun Lee [2], Changhyun Pang [3], Ki Sung Kang [1,*] and Ki Hyun Kim [2,*]

1 College of Korean Medicine, Gachon University, Seongnam 13120, Republic of Korea
2 School of Pharmacy, Sungkyunkwan University, Suwon 16419, Republic of Korea
3 School of Chemical Engineering, Sungkyunkwan University, Suwon 16419, Republic of Korea
* Correspondence: kkang@gachon.ac.kr (K.S.K.); khkim83@skku.edu (K.H.K.);
Tel.: +82-31-750-5402 (K.S.K.); +82-31-290-7700 (K.H.K.)
† These authors contributed equally to this study.

Supporting Information Contents:

Figure S1. The ^{1}H NMR spectrum of **1** (CD_3OD, 800 MHz)……S3
Figure S2. The ^{13}C NMR spectrum of **1** (CD_3OD, 200 MHz)……S4
Figure S3. HPLC chromatogram and UV and MS data of LC/MS for compound **1**……S5
Figure S4. The ^{1}H NMR spectrum of **2** (CD_3OD, 800 MHz)……S6
Figure S5. HPLC chromatogram and UV and MS data of LC/MS for compound **2**……S7
Figure S6. The ^{1}H NMR spectrum of **3** (CD_3OD, 800 MHz)……S8
Figure S7. The ^{13}C NMR spectrum of **3** (CD_3OD, 200 MHz)……S9
Figure S8. HPLC chromatogram and UV and MS data of LC/MS for compound **3**……S10
Figure S9. The ^{1}H NMR spectrum of **4** (CD_3OD, 800 MHz)……S11
Figure S10. HPLC chromatogram and UV and MS data of LC/MS for compound **4**……S12
Figure S11. The ^{1}H NMR spectrum of **5** (CD_3OD, 800 MHz)……S13
Figure S12. HPLC chromatogram and UV and MS data of LC/MS for compound **5**……S14

Figure S1. The ^{1}H NMR spectrum of **1** (CD_3OD, 800 MHz)

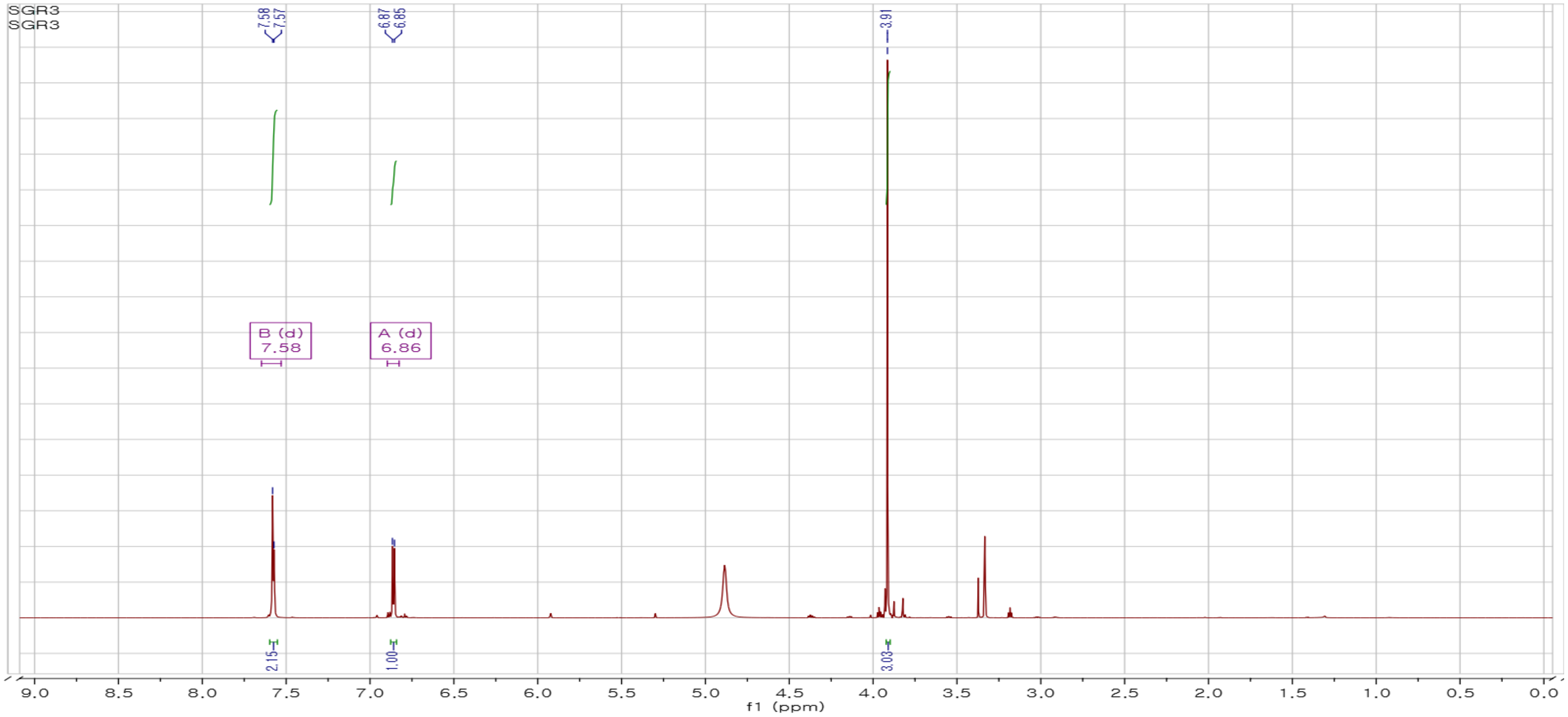

Figure S2. The ^{13}C NMR spectrum of **1** (CD_3OD, 200 MHz)

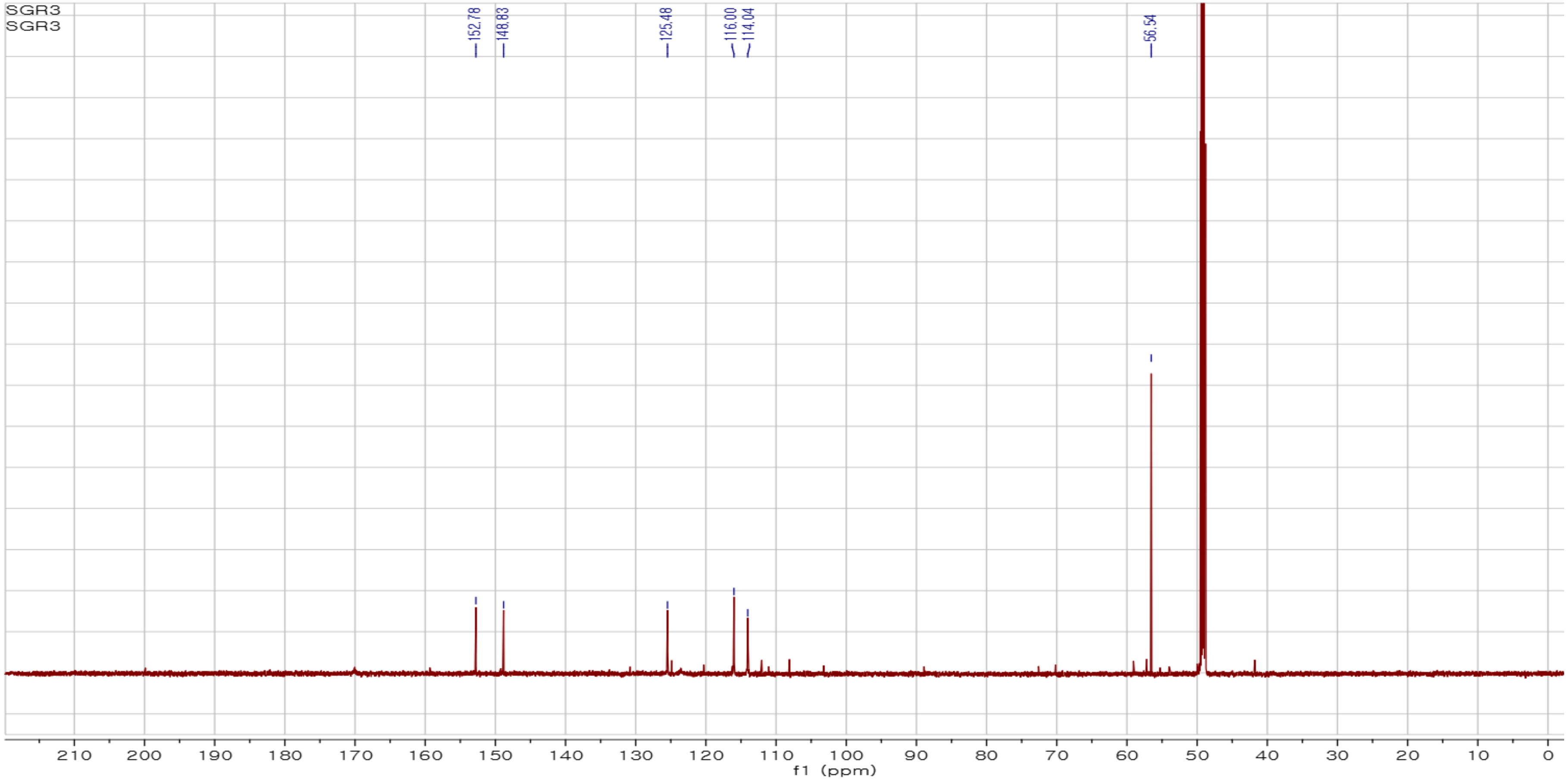

Figure S3. (A) HPLC chromatogram (detection wavelength was set as 254 nm) and (B) UV and MS data of LC/MS for compound **1**.

(A)

(B)

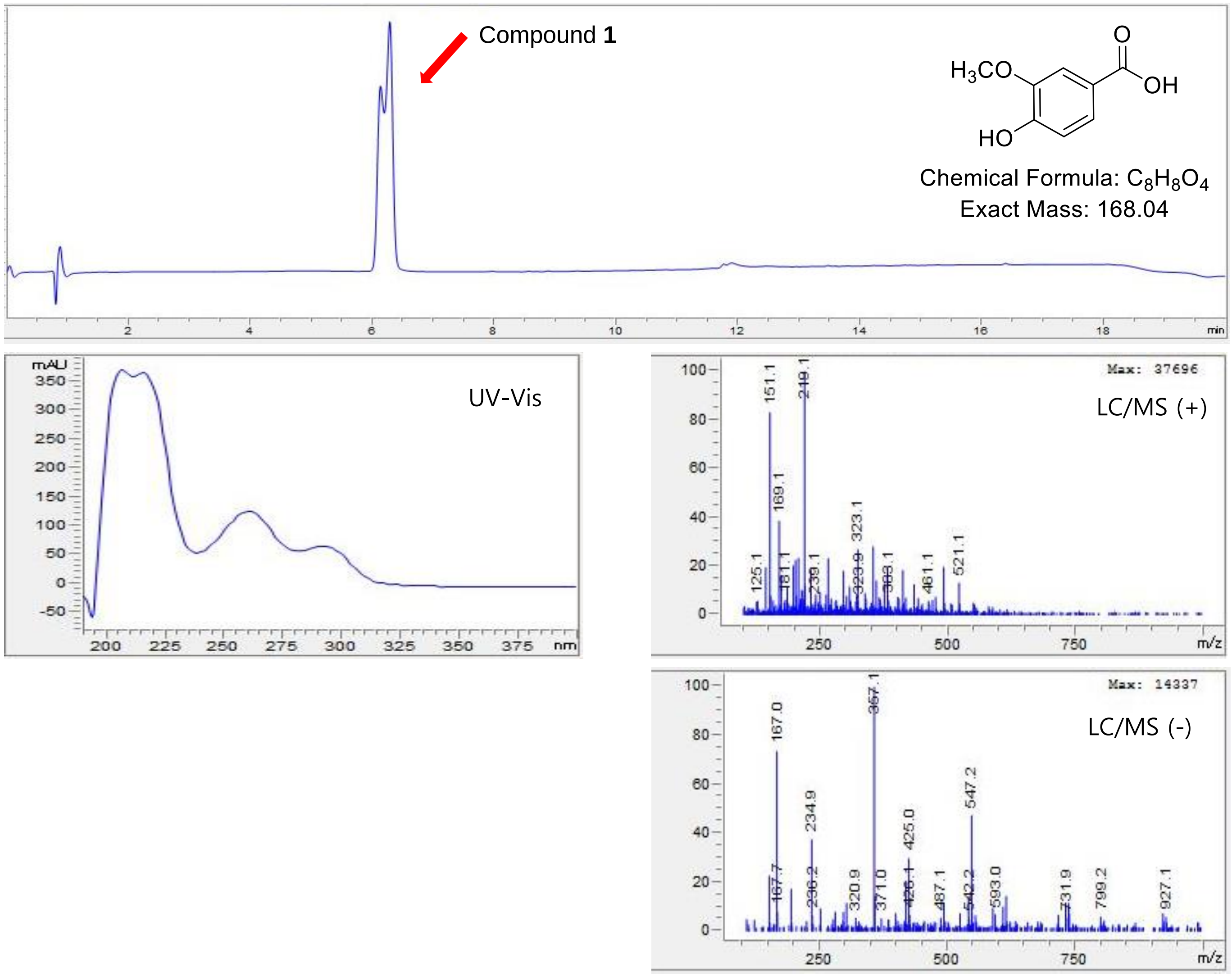

Figure S4. The ^{1}H NMR spectrum of **2** (CD_3OD, 800 MHz)

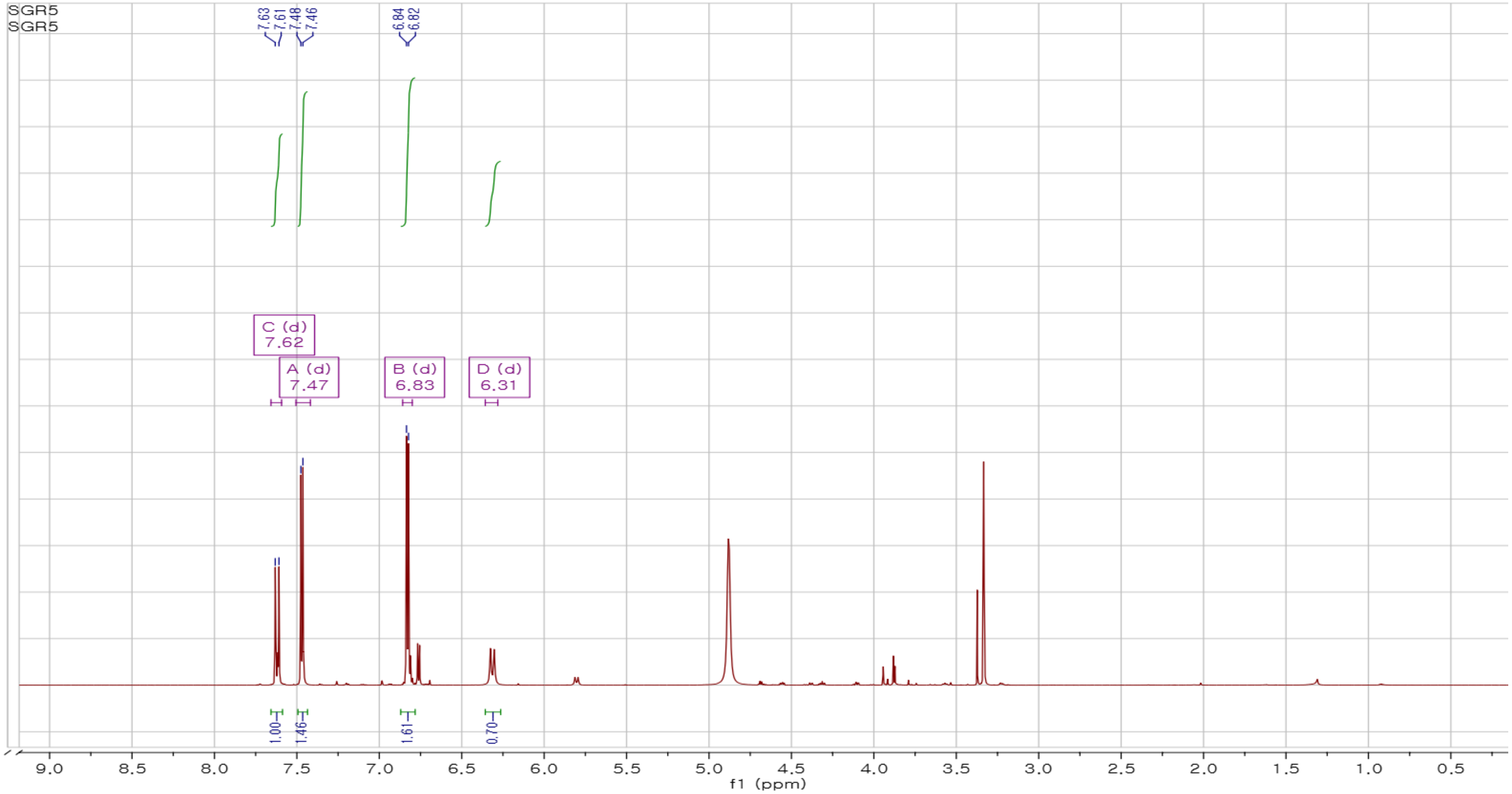

Figure S5. (A) HPLC chromatogram (detection wavelength was set as 254 nm) and (B) UV and MS data of LC/MS for compound **2**

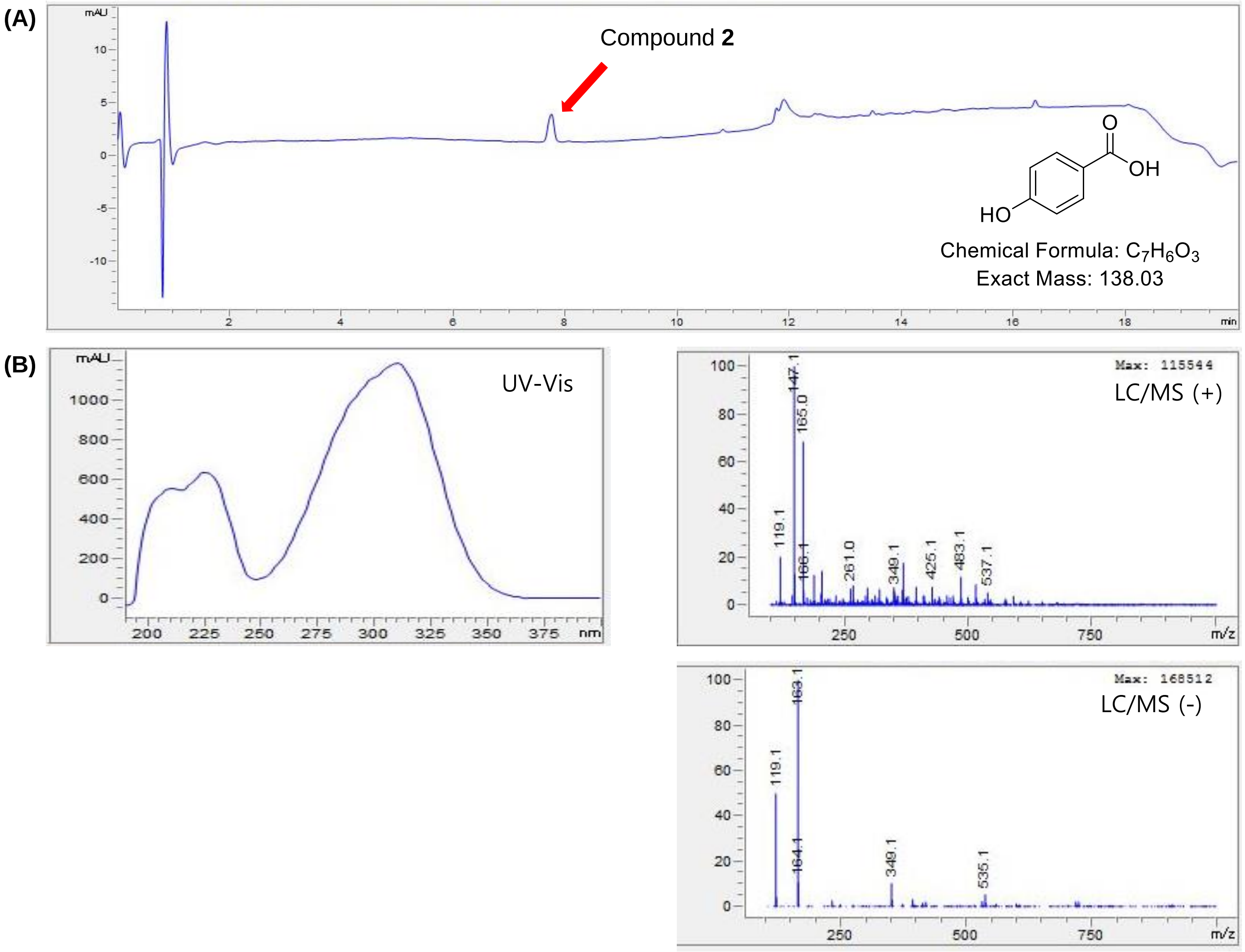

Figure S6. The ^{1}H NMR spectrum of **3** (CD_3OD, 800 MHz)

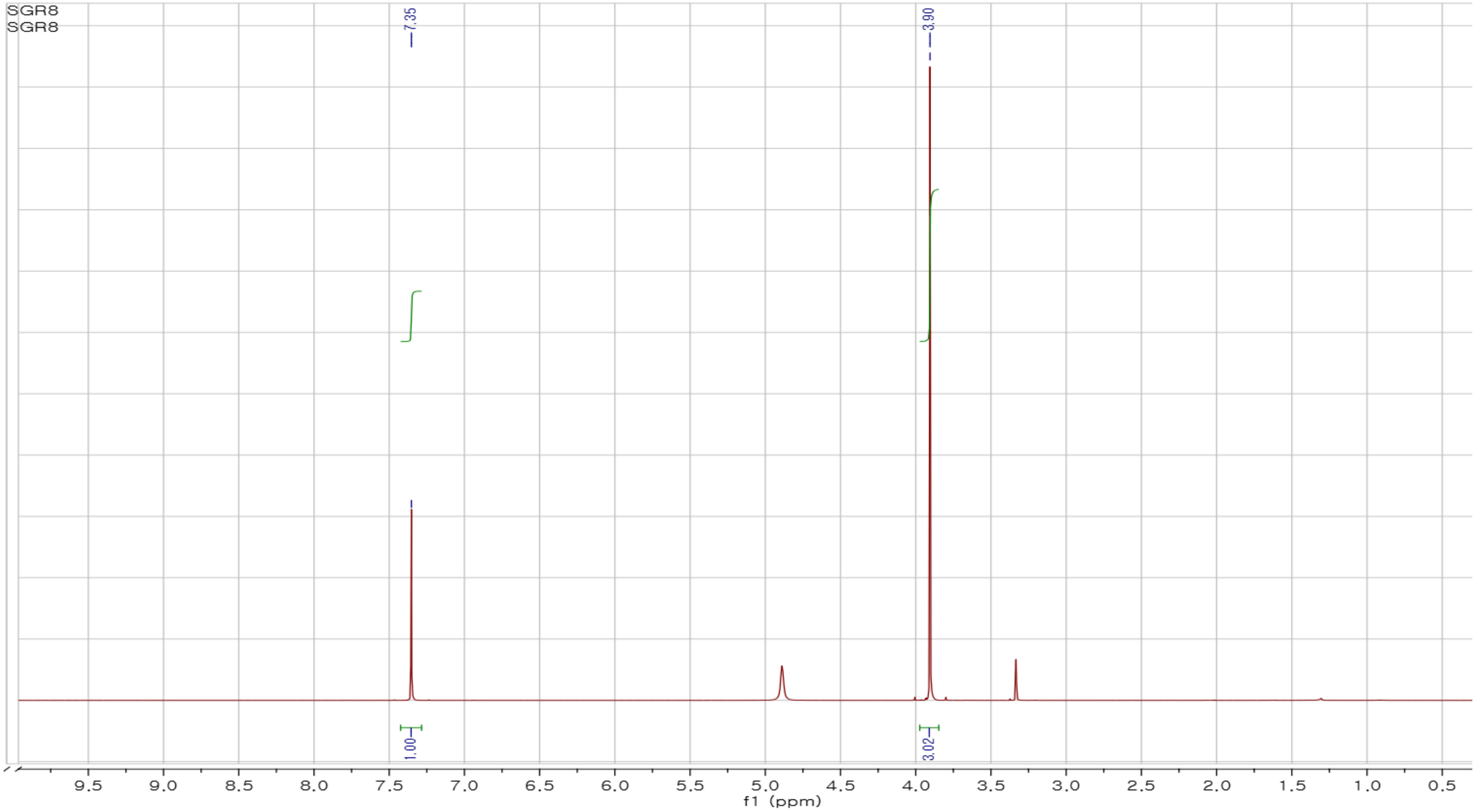

Figure S7. The ^{13}C NMR spectrum of **3** (CD_3OD, 200 MHz)

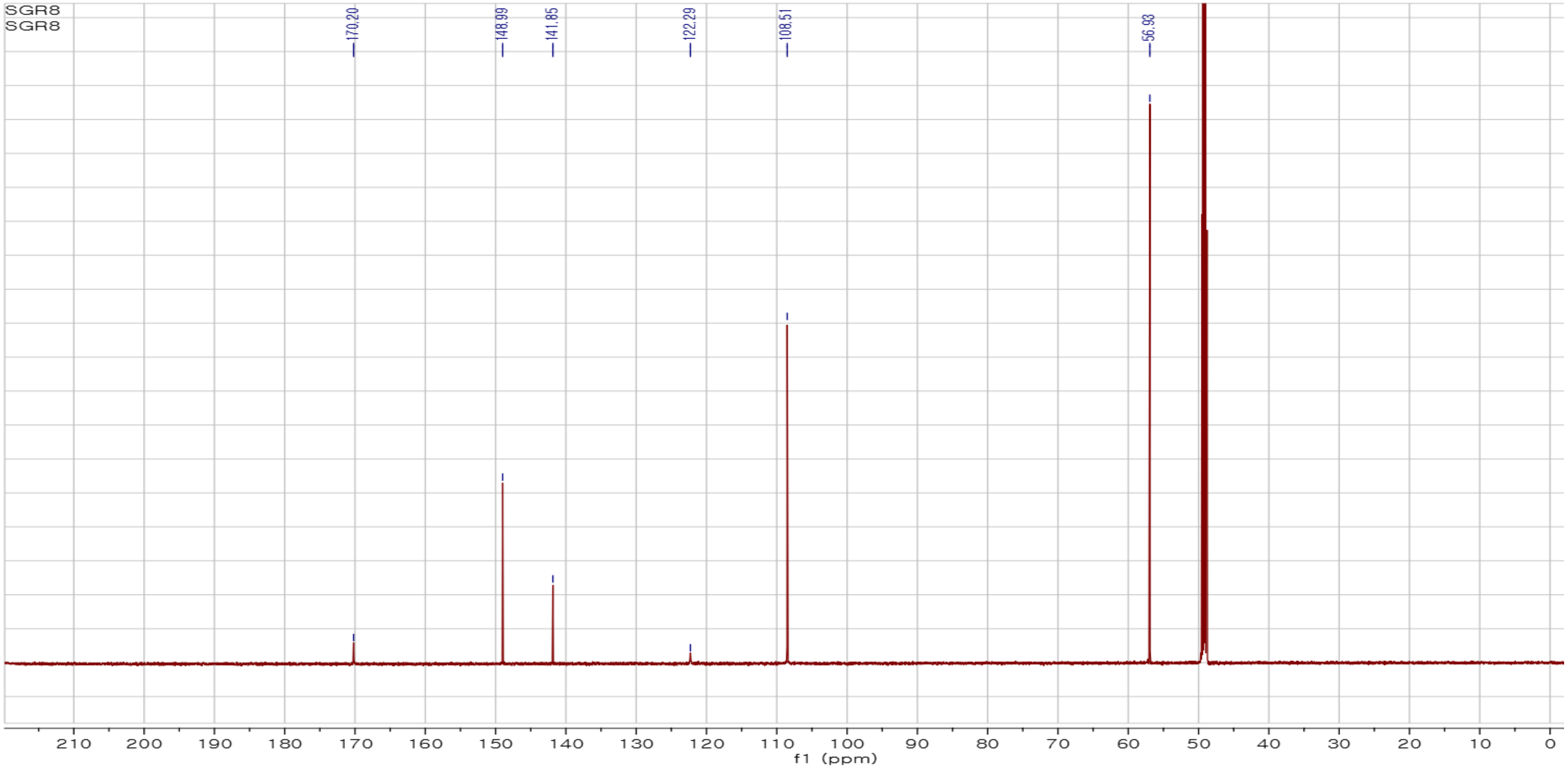

Figure S8. (A) HPLC chromatogram (detection wavelength was set as 254 nm) and (B) UV and MS data of LC/MS for compound **3**

(A)

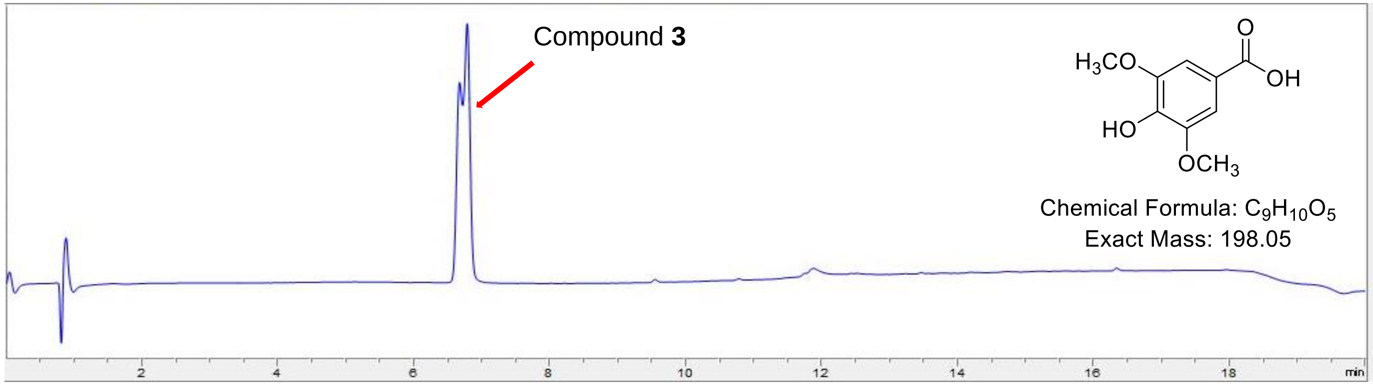

(B)

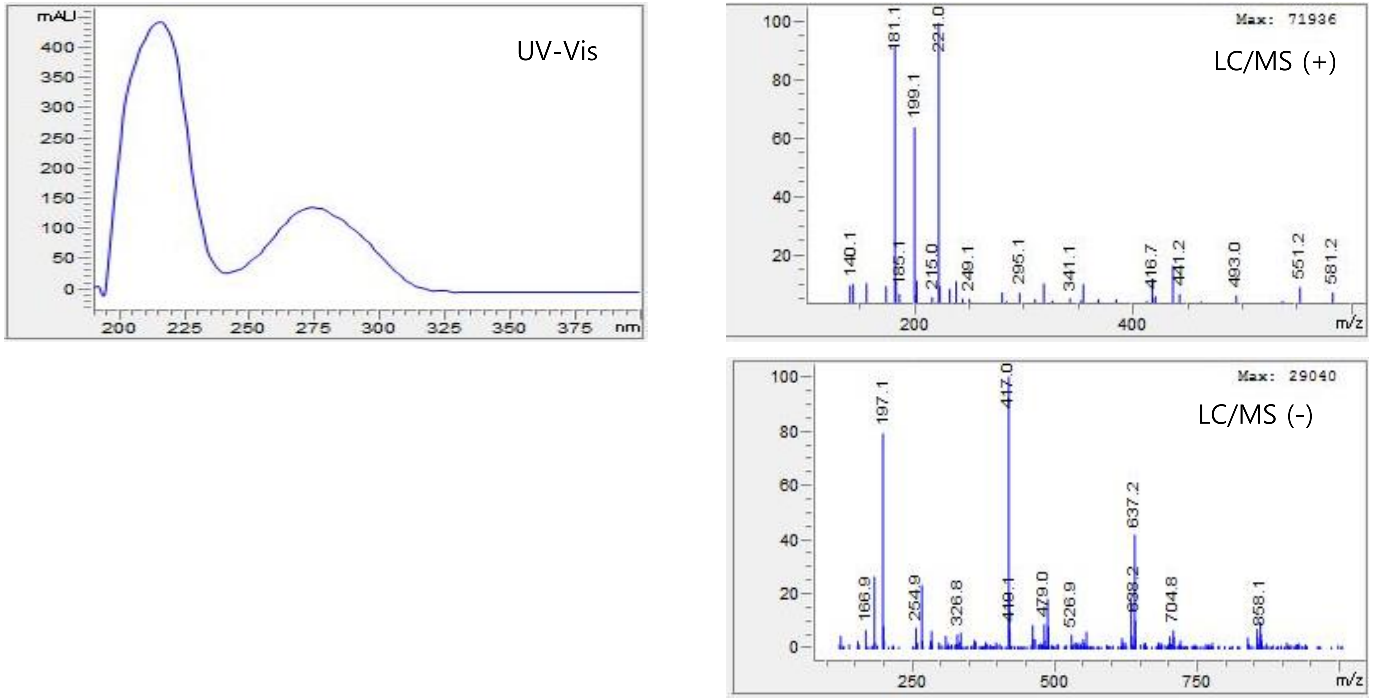

Figure S9. The ^{1}H NMR spectrum of **4** (CD_3OD, 800 MHz)

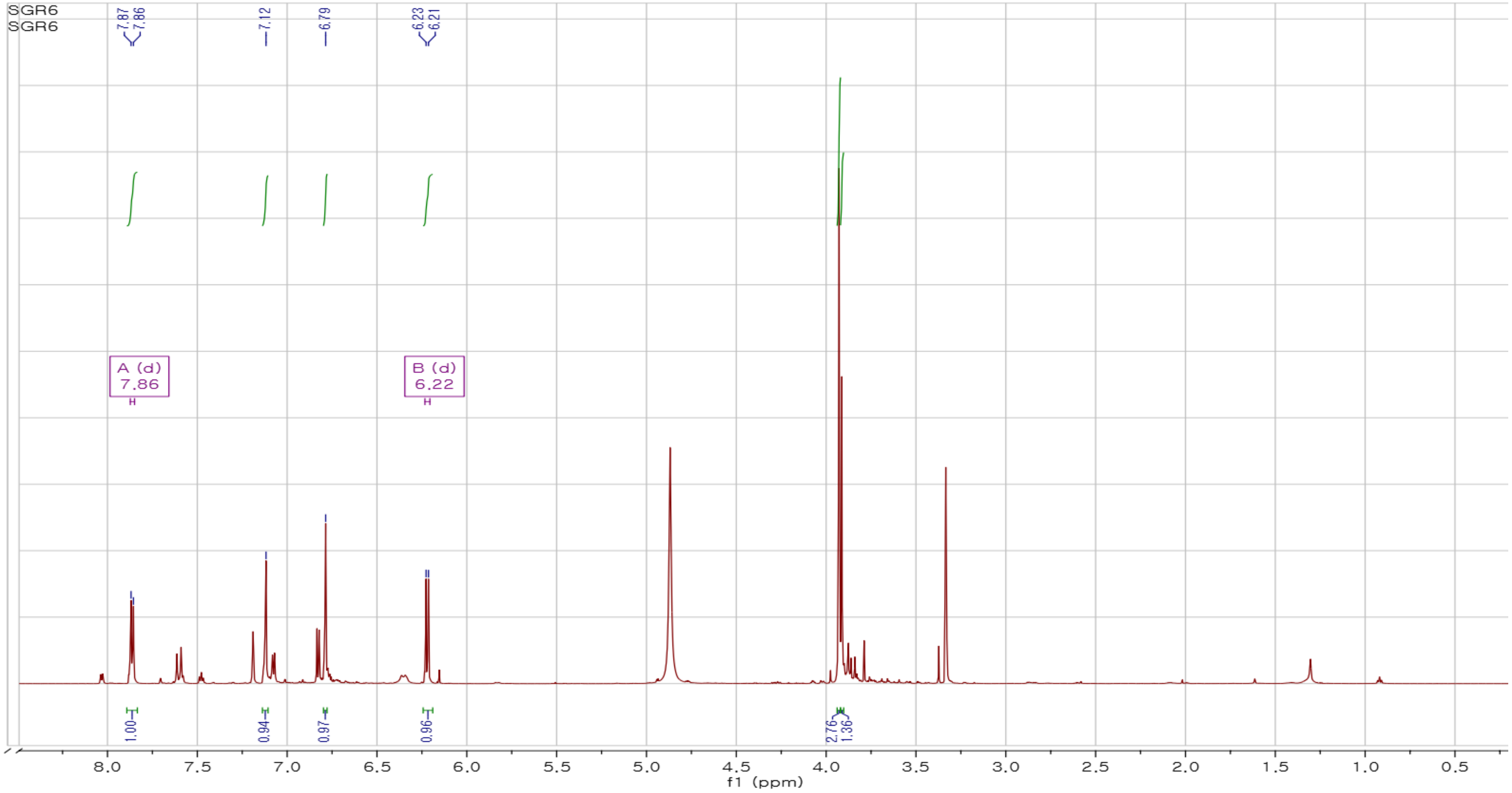

Figure S10 (A) HPLC chromatogram (detection wavelength was set as 254 nm) and (B) UV and MS data of LC/MS for compound **4**

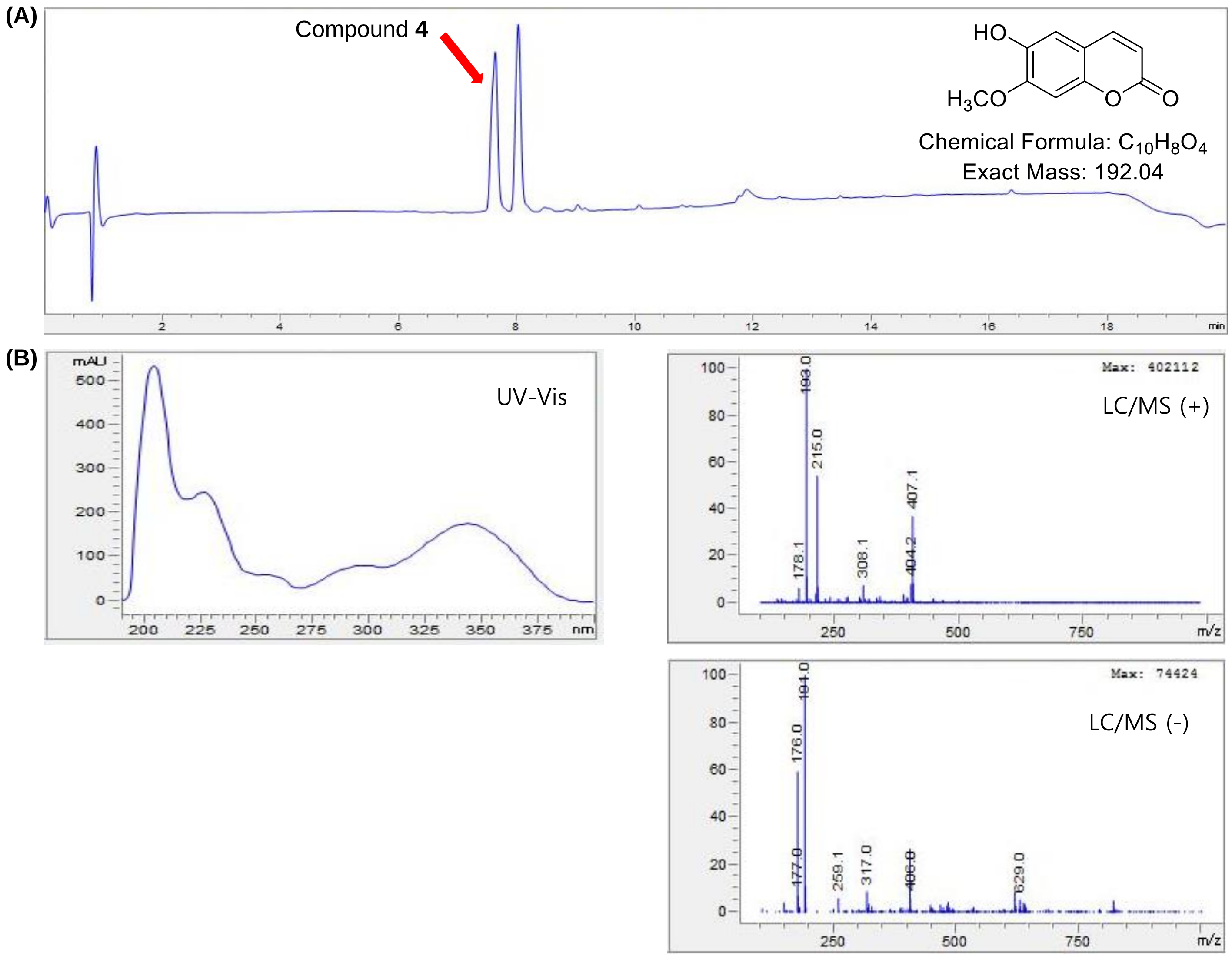

Figure S11. The ^{1}H NMR spectrum of **5** (CD_3OD, 800 MHz)

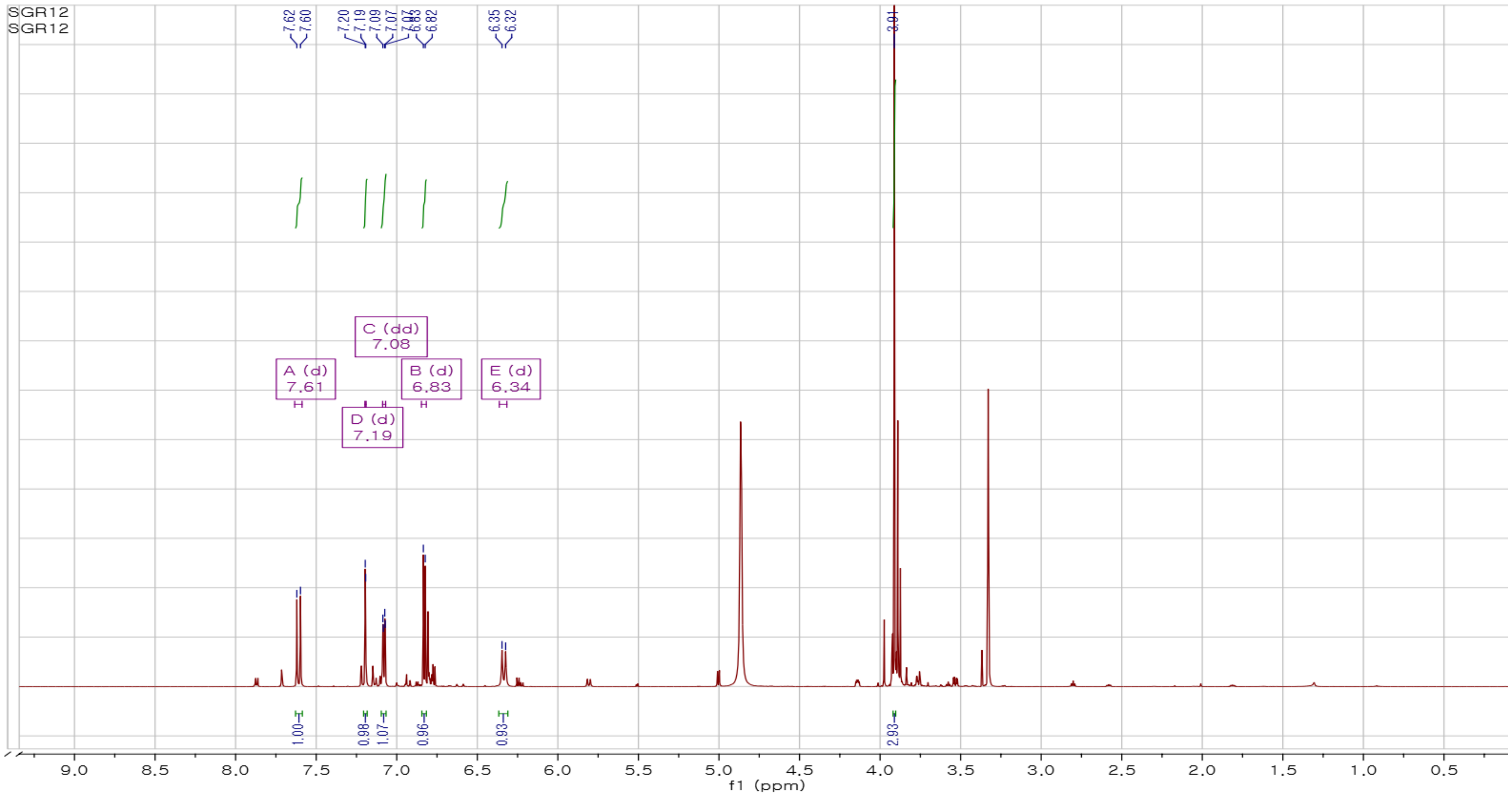

Figure S12 (A) HPLC chromatogram (detection wavelength was set as 254 nm) and (B) UV and MS data of LC/MS for compound **5**

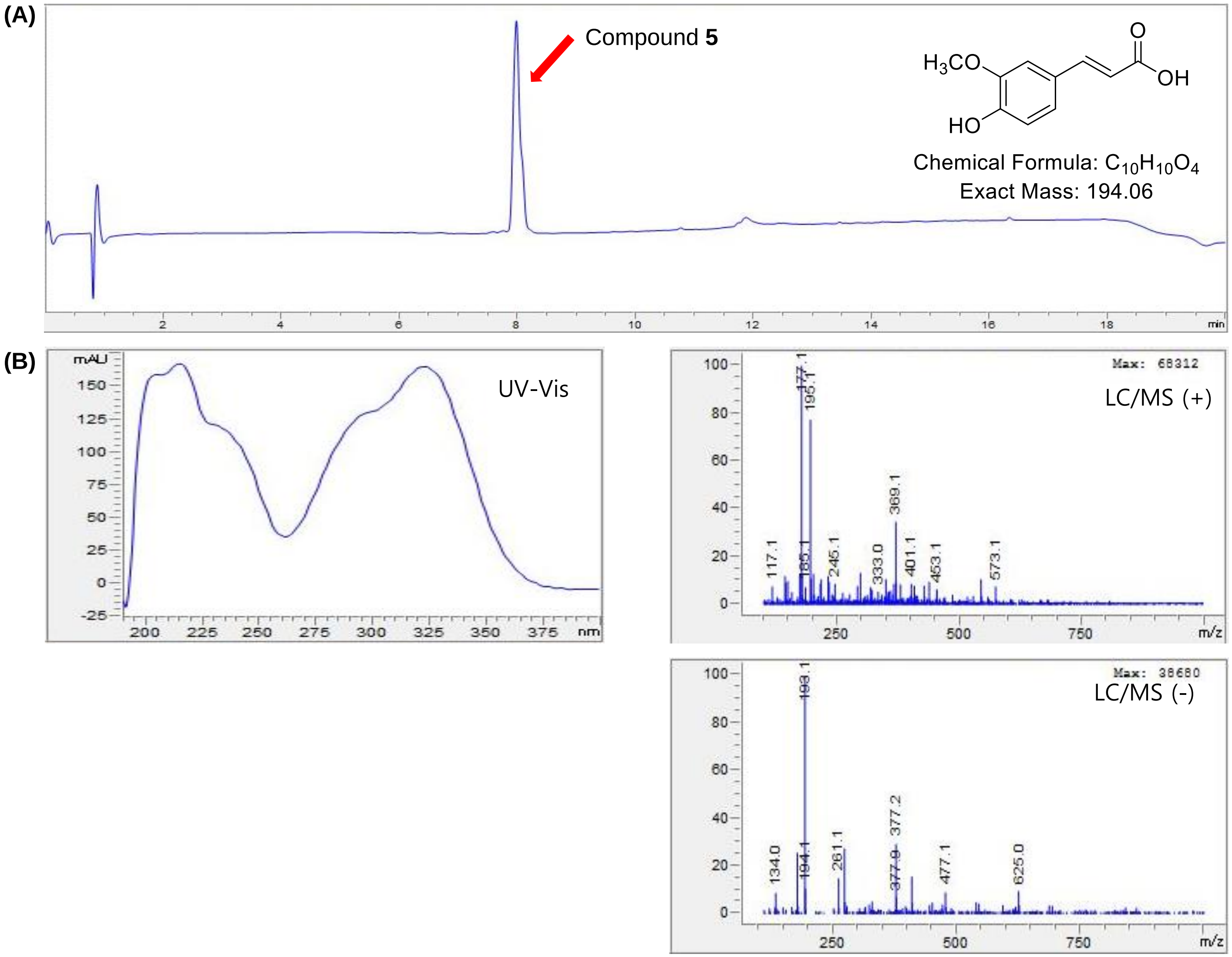

General Experimental Procedure

Electrospray ionization (ESI) and HR-ESI mass spectra were recorded on a Waters Micromass Q-TofUltima ESI-TOF mass spectrometer (Waters, New York, NY, USA). NMR spectra were recorded on a Varian UNITY INOVA 700 NMR spectrometer operating at 800 MHz (^{1}H) and 200 MHz (^{13}C), with chemical shifts in ppm (δ). Preparative HPLC was performed using a Waters 1525 Binary HPLC pump with a Waters 996 Photodiode Array Detector (Waters Corporation, Milford, CT, USA). Semi-preparative HPLC was performed on a Shimadzu Prominence HPLC System with SPD-20A/20AV Series Prominence HPLC UV-Vis Detectors (Shimadzu, Tokyo, Japan). LC/MS analysis was conducted on an Agilent 1200 Series HPLC system (Agilent Technologies, Santa Clara, CA, USA) equipped with a diode array detector and a 6130 Series ESI mass spectrometer using analytical Kinetex (4.6 × 100 mm, 3.5 μm). Silica gel 60 (70–230 mesh and 230–400 mesh; Merck) and RP-C_{18} silica gel (40–63 μm; Merck) were used for column chromatography. Sephadex LH-20 (Pharmacia, Uppsala, Sweden) was used as the packing material for molecular sieve column chromatography. Precoated silica gel F_{254} and RP-18 F_{254s} plates (Merck, Darmstadt, Germany) were used for thin-layer chromatography (TLC). Spots were detected by TLC under UV light or by heating after spraying with anisaldehyde-sulfuric acid.

Plant Material

Bark samples of *A. tegmentosum* were obtained from Hongcheon and Jeongseon in the Gangwon Province, Korea, in June 2013. The material was confirmed by one of the authors (K.H.K.). A voucher specimen (MIMH-35) was deposited in the herbarium of Hongcheon Institute of Medicinal Herb, Hongcheon, Korea.

Cell Culture

ER-positive MCF-7 human breast epithelial cell line (American Type Culture Collection, Manassas, VA, USA) was cultured in the Roswell Park Memorial Institute (RPMI)-1640 medium (Cellgro, Manassas, VA, USA) supplemented with 100 µg/mL streptomycin, 100 U/mL penicillin, and 10% fetal bovine serum (Gibco BRL, Grand Island, NY, USA). MCF-7 cells were stored at 37 °C in an incubator at a CO_2 concentration of approximately 5%.

Western Blotting Analysis

MCF-7 cells were seeded into a 60 mm culture plate at a density of 2×10^5 cells. After incubating for 24 h, the cells were treated with the samples in the treatment medium, as described previously. Fresh treatment medium was replaced after 48 h and the treatment was continued for 96 h. The treatment medium was discarded, and the cells were washed with Dulbecco's phosphate-buffered saline (Welgene Inc., Daegu, Korea). Next, the cells were harvested and lysed in 1× radioimmunoprecipitation assay buffer (Tech & Innovation, Gangwon, Korea) supplemented with a proteinase inhibitor cocktail (Roche Diagnostics, Basel, Switzerland) to obtain whole-cell extracts. Proteins in the cell extracts of each sample were quantified using a Pierce BCA Protein Assay Kit (Thermo Fisher Scientific, Waltham, MA, USA). Equal amounts of proteins (20 µg/lane) were separated using 10% sodium dodecyl sulfate-polyacrylamide gel electrophoresis at 80 V, 400 mA until the bands were separated and transferred onto polyvinylidene difluoride membranes. The membranes were blocked with 5% skim milk in Tris-buffered saline (1×) for 1 h, and the separated proteins were identified via incubation with epitope-specific primary and secondary antibodies (Cell Signaling Technology, Danvers, MA, USA). The membranes were visualized using the SuperSignal West Femto Maximum Sensitivity Substrate (Thermo Fisher Scientific) and imaged using the FUSION Solo Chemiluminescence System (Vilber Lourmat Deutschland GmbH; Eberhardzell, Germany).

Statistical Analysis

All assays were performed in triplicate and repeated at least thrice. All data are presented as the mean ± standard error of the mean. Statistical significance was determined using one-way analysis of variance and multiple comparisons with Tukey's post-hoc test. Statistical significance was set at $p < 0.05$. All analyses were performed using Prism ver. 8.1 (GraphPad Software, San Diego, CA, USA).
